# Profiles of autism characteristics in thirteen genetic syndromes: a machine learning approach

**DOI:** 10.1186/s13229-022-00530-5

**Published:** 2023-01-13

**Authors:** Natali Bozhilova, Alice Welham, Dawn Adams, Stacey Bissell, Hilgo Bruining, Hayley Crawford, Kate Eden, Lisa Nelson, Christopher Oliver, Laurie Powis, Caroline Richards, Jane Waite, Peter Watson, Hefin Rhys, Lucy Wilde, Kate Woodcock, Joanna Moss

**Affiliations:** 1 School of Psychology, University of Surrey, Guilford, UK; 2 School of Psychology, University of Leicester, Leicester, UK; 3 Autism Centre of Excellence, Griffith University, Brisbane, Australia; 4 School of Psychology, University of Birmingham, Edgbaston, UK; 5 Department of Child and Adolescent Psychiatry, Amsterdam UMC, University of Amsterdam, Amsterdam, The Netherlands; 6 Mental Health and Wellbeing Unit, Warwick Medical School, University of Warwick, Coventry, UK; 7 School of Psychology, College of Health and Life Sciences, Aston University, Birmingham, UK; 8 MRC Brain and Cognition Unit, University of Cambridge, Cambridge, UK; 9 Francis Crick Institute, London, UK; 10 School of Psychology, Open University, Milton Keynes, UK

**Keywords:** Autism, Genetic syndromes, SVM, Machine learning, Behavioural phenotype

## Abstract

**Background:**

Phenotypic studies have identified distinct patterns of autistic characteristics in genetic syndromes associated with intellectual disability (ID), leading to diagnostic uncertainty and compromised access to autism-related support. Previous research has tended to include small samples and diverse measures, which limits the generalisability of findings. In this study, we generated detailed profiles of autistic characteristics in a large sample of > 1500 individuals with rare genetic syndromes.

**Methods:**

Profiles of autistic characteristics based on the Social Communication Questionnaire (SCQ) scores were generated for thirteen genetic syndrome groups (Angelman *n* = 154, Cri du Chat *n* = 75, Cornelia de Lange *n* = 199, fragile X *n* = 297, Prader–Willi *n* = 278, Lowe *n* = 89, Smith–Magenis *n* = 54, Down *n* = 135, Sotos *n* = 40, Rubinstein–Taybi *n* = 102, 1p36 deletion *n* = 41, tuberous sclerosis complex *n* = 83 and Phelan–McDermid *n* = 35 syndromes). It was hypothesised that each syndrome group would evidence a degree of specificity in autistic characteristics. To test this hypothesis, a classification algorithm via support vector machine (SVM) learning was applied to scores from over 1500 individuals diagnosed with one of the thirteen genetic syndromes and autistic individuals who did not have a known genetic syndrome (ASD; *n* = 254). Self-help skills were included as an additional predictor.

**Results:**

Genetic syndromes were associated with different but overlapping autism-related profiles, indicated by the substantial accuracy of the entire, multiclass SVM model (55% correctly classified individuals). Syndrome groups such as Angelman, fragile X, Prader–Willi, Rubinstein–Taybi and Cornelia de Lange showed greater phenotypic specificity than groups such as Cri du Chat, Lowe, Smith–Magenis, tuberous sclerosis complex, Sotos and Phelan-McDermid. The inclusion of the ASD reference group and self-help skills did not change the model accuracy.

**Limitations:**

The key limitations of our study include a cross-sectional design, reliance on a screening tool which focuses primarily on social communication skills and imbalanced sample size across syndrome groups.

**Conclusions:**

These findings replicate and extend previous work, demonstrating syndrome-specific profiles of autistic characteristics in people with genetic syndromes compared to autistic individuals without a genetic syndrome. This work calls for greater precision of assessment of autistic characteristics in individuals with genetic syndromes associated with ID.

**Supplementary Information:**

The online version contains supplementary material available at 10.1186/s13229-022-00530-5.

## Background

Autism is highly heritable [1] and characterised by a range of behavioural difficulties or differences in three core domains: social interactions, communication and restricted, repetitive behaviours/interests [2, 3]; although current diagnostic frameworks group social interaction and communication into a single domain [2]. Despite discretely defined diagnostic categories, there is substantial genotypic [4] and phenotypic variability across autistic individuals.

To understand this variability, the ‘fractionation of the triad’ theory [3] inspired a line of research, which identified weak-to-moderate behavioural [5–7] and genetic [8] correlations between the three domains. Divergent developmental trajectories have also been described for each domain [9], and the presence of difficulties or differences across *all* domains have only been identified in a quarter of the population [9]. These findings, alongside data which strongly point towards the existence of distinct biological influences for each domain [3, 10–12], suggest that heterogeneity across autistic people is present at the cognitive, behavioural and biological level [13]. To understand this complexity and advance understanding of phenotypic heterogeneity in autism, research has increasingly focused on individuals with genetic syndromes, in which phenotypic heterogeneity of autistic characteristics is frequently described [14].

Individuals with genetic syndromes associated with intellectual disability (ID) are more likely to evidence autistic characteristics compared to individuals in the general population [15, 16]. While specific prevalence rates of autistic characteristics are variable across different genetic syndrome groups, robust evidence demonstrates increased prevalence of autistic characteristics across this population. Based on a recent population-based study, 10–18% of autistic individuals also have a co-occurring ID, often as part of a known genetic syndrome [17]. However, individuals with genetic syndromes often demonstrate syndrome-associated autism profiles, highlighting the presence of subtle quantitative and qualitative differences in phenotypic expression of autism [18–24]. It has been suggested that these cross-syndrome phenotypic differences reflect ‘milder’ manifestations of autistic characteristics in genetic syndromes compared to non-syndromic autism [21]. This explanation is insufficient and potentially harmful given that the presentation of autistic traits is atypical rather than ‘milder’ and there is no evidence to support that such presentations of autistic traits are less clinically and functionally impactful for individuals relative to the profile of autistic characteristics typically associated with non-syndromic autism.

Within-syndrome phenotypic variability and cross-syndrome phenotypic similarities have also been documented [25]. For example, different genetic syndromes evidence an overlap in autism-related phenotypic expression [15, 25–27], suggesting that different genetic syndromes might be associated with distinct, but also partially shared autism profiles. This vast variability in autism phenotypic expression between and within genetic syndromes coupled with ID predisposes to high rates of misdiagnosis or diagnostic overshadowing [28]. Detailed descriptions of autism-related profiles across genetic syndromes could lead to the development of more sensitive and specific autism and related assessments and further encourage individualised approaches to autism screening and diagnostic assessments for individuals with complex clinical needs.

Using an established support vector machine (SVM) learning approach, previously adopted by Bruining et al. [29] and more recently by Lee et al. [24], we generated fine-grained descriptions of autistic characteristics in individuals with one of the thirteen genetic syndromes. *Aim 1:* The primary aim of this study was to replicate and extend previously reported ‘behavioural signatures’ (interchangeably referred as ‘profiles’ in this study) of autism in individuals with rare genetics syndromes [29] in a considerably larger sample (*n* = 1582) including both adults and children with thirteen genetic syndromes and autistic people without a genetic syndrome. *Aim 2:* To understand whether and how much variability in adaptative behaviour skills contributed to the generation of these behavioural signatures. *Aim 3:* To test whether misclassified individuals with genetic syndromes were alternatively classified into another syndrome group or the autistic group. *Aim 4:* To understand which items on the Social Communication Questionnaire were more or less likely to contribute to the specific signatures within each group.

## Method

### Participants

This study used retrospective baseline data from one of the largest cross-syndrome databases in the UK (held at a UK-based university). The total sample included 1702 individuals with genetic syndromes associated with ID and 264 autistic individuals with varying levels of adaptive skills. The database was first set up in 2003, and the last follow-up was completed in 2018. The first wave of data collection included eight behavioural and health measures as well as diagnostic information (i.e., presence/absence of a genetic syndrome). As part of the follow-ups, more measures and groups (including austistic individuals without a genetic syndrome) were added to the database. Currently, this database represents the largest longitudinal data on individuals with genetic syndromes associated with ID in the UK.

Each of the thirteen genetic syndromes was included in this paper due to their reported increased likelihood of autism compared to the general population [16]. We also used opportunity sampling based on the data available at the time of analysis. In total, 1582 individuals with genetic syndromes and 258 autistic individuals who did not have a known genetic syndrome, all over four years of age, were included in the analysis. The genetic syndrome groups had varying sample sizes and included: Angelman (AS, *n* = 154), Cri du Chat (CdCS, *n* = 75), Cornelia de Lange (CdLS, *n* = 199), fragile X (FXS, *n* = 297), Prader–Willi (PWS, *n* = 278), Lowe (LS, *n* = 89), Smith–Magenis (SMS, *n* = 54), Down (DS, *n* = 135), Sotos (SS, *n* = 40), Rubinstein–Taybi (RTS, *n* = 102), 1p36 deletion (*n* = 41), Phelan-McDermid (PMS, *n* = 35) syndromes and tuberous sclerosis complex (TSC, *n* = 83). Individuals in these groups were included in the analysis irrespective of the presence or absence of an autism diagnosis.

Due to missing data (> 30%) or unsuitable age for assessment of social and communication skills with the SCQ (3 years or younger), 120 individuals with genetic syndromes (AS *n* = 3; FXS *n* = 21; PWS *n* = 25; RTS *n* = 3; CdLS *n* = 25; DS *n* = 9; CdCS *n* = 6; 1p36 *n* = 6; LS *n* = 7; SMS *n* = 6; TSC *n* = 4; SS *n* = 10; PMS *n* = 1) and 6 autistic individuals without a genetic syndrome were excluded from the study. For demographic characteristics of the entire sample, refer to Table 1.

**Table 1:** Sample description of the thirteen genetic syndromes and the autistic group

	*N*	Gender	Age		Social Communication SCQ			Adaptive functioning Wessex Behaviour Scale					
	Total	Female	Years (M ± SD)	Range	ASD cut-off %	Total (M ± SD)	Hearing %	Vision %	Speech %	Mobility %		Self-help %	
							Typical	Typical	Verbal	Typical	M ± SD	Typical	M ± SD
AS	154	73	12.74 ± 8.74	4–52	66	17.13 ± 5.28	98	84	32	50	4.73 ± 1.51	30	4.74 ± 1.21
FXS	297	0	16.90 ± 9.91	4–55	77	20.59 ± 6.82	97	88	91	74	5.51 ± 0.98	91	7.40 ± 1.38
PWS	278	111	15.92 ± 10.39	4–49	45	14.49 ± 7.12	91	64	93	67	5.52 ± 0.94	90	7.66 ± 1.33
RTS	102	43	18.62 ± 10.91	4–49	60	16.64 ± 5.34	84	85	84	73	5.31 ± 1.27	75	6.47 ± 1.58
CdLS	199	110	17.33 ± 9.76	4–45	71	20.20 ± 6.52	64	68	61	60	5.20 ± 1.16	54	5.80 ± 1.96
DS	135	78	23.73 ± 12.80	4–62	16	10.01 ± 7.01	64	62	95	93	5.87 ± 0.51	94	7.94 ± 1.39
CdCS	75	46	16.07 ± 11.97	4–44	41	14.63 ± 6.29	85	85	73	50	4.86 ± 1.38	55	5.54 ± 1.60
1p36	41	28	9.62 ± 7.66	4–39	64	18.89 ± 7.02	64	41	47	58	4.25 ± 1.61	36	4.91 ± 1.82
LS	89	0	16.21 ± 10.25	4–51	73	18.77 ± 6.78	97	15	86	52	4.86 ± 1.41	66	6.11 ± 1.73
SMS	54	30	15.76 ± 9.99	4–46	56	18.22 ± 7.08	59	67	93	76	5.52 ± 0.93	76	6.65 ± 1.82
TSC	83	36	19.21 ± 10.80	4–50	70	20.01 ± 7.94	96	89	76	76	5.37 ± 1.10	67	6.74 ± 2.16
SS	35	13	16.22 ± 9.17	4–43	69	17.84 ± 8.00	81	75	97	92	5.70 ± 0.97	89	7.24 ± 1.61
PMS	40	18	11.31 ± 8.14	4–37	75	21.55 ± 7.21	90	80	40	77	5.29 ± 1.35	30	4.84 ± 1.19
Total	1562	586	16.82 ± 10.58	4–62	58	17.47 ± 7.41	84	72	78	69	5.32 ± 1.18	73	6.64 ± 1.88
ASD	256	36	11.71 ± 6.28	4–46	100	26.43 ± 5.42	97	96	93	94	5.87 ± 0.63	90	7.47 ± 1.48

This table reflects the total sample size of each syndrome group prior to the exclusion of participants due to technical or other reasons. The term ‘typical’ has been used to replace the original questionnaire term ‘normal’, in keeping with current terminology

*AS* Angelman syndrome, *FXS* fragile X syndrome, *PWS* Prader–Willi syndrome, *RTS* Rubinstein–Taybi syndrome, *CdLS* Cornelia de Lange syndrome, *DS* Down syndrome, *CdCS* Cri du Chat syndrome, *1p36* 1p6 deletion syndrome, *LS* Lowe syndrome, *SMS* Smith–Magenis syndrome, *SS* Sotos syndrome, *TSC* Tuberous sclerosis complex, *PMS* Phelan-McDermid syndrome, *ASD* Autism spectrum disorder. *Total* All individuals with genetic syndromes, *SCQ* Social Communication Questionnaire

### Recruitment

Potential participants and their parents/carers were invited to take part in a questionnaire survey evaluating the behavioural characteristics associated with a range of genetic syndromes. Questionnaire responses were collected from 2003 to 2018. Participants were recruited via syndrome support groups/associations (e.g., Fragile X Society UK, CdLS Foundation UK and Ireland and the National Autistic Society). The recruitment strategy was agreed between the former research centre and the relevant associations/charities to maximise recruitment success and minimise potential burden on the participants. Favourable ethical approval was granted by the Coventry Research Ethics Committee (REC, 10/H1210/1), and the current study underwent institutional governance review. Individuals with genetic syndromes were included in the study if they reported receiving a diagnosis of the genetic syndrome from an appropriate professional (i.e., a paediatrician, clinical geneticist, general practitioner, psychiatrist, or neurologist). Parents and caregivers were also invited to share genetic confirmation letters (where such a record of genetic information was available, and families consented to genetic confirmation sharing). Autistic individuals without a genetic syndrome were included in the analysis if they reached the suggested threshold for autism or autism spectrum disorder (ASD) on the SCQ*,* indicated that an autism diagnosis had been made by an appropriate professional (i.e., these participants had received a diagnosis of autism from a clinical psychologist, psychiatrist, educational psychologist, speech and language therapist, paediatrician, general practitioner) *and* confirmed the absence of a genetic syndrome diagnosis.

Inclusion criteria for the current study included: (1) presence of a rare genetic syndrome or autism, or both, (2) age 4 years or older, (3) an ability of the caregiver/child/adult to provide informed consent or assent to participate in the study as appropriate to their capacity to consent/assent, (4) the informant/participant should be fluent in English. All participants who met the inclusion criteria outlined above were included in the analysis.

### Measures

#### Social Communication Questionnaire (SCQ) [30]

The SCQ is a widely used screening tool, which focuses on autistic characteristics [30]. This parent/caregiver-report questionnaire is based on the Autism Diagnostic Interview- Revised (ADI-R), which is a well-established diagnostic interview [31]. The SCQ has also been used for understanding autism-related behavioural phenotypes in populations with genetic syndromes and genetic population studies of autism [15].

The SCQ consists of 40 items with a binary response (yes/no). The measure is suitable for individuals who are 4 years or older. There are two versions of the SCQ, lifetime and current. The lifetime version assesses the entire developmental history of the participant, which is used to support diagnostis or to indicate that a diagnosis should be considered. The current version focuses on the participant’s behaviour in the past 3 months, which is suitable for assessing current autistic traits for support and educational plans. SCQ items are scored as 0 and 1; 0 reflects an absence of the relevant behaviour, and 1 reflects the presence of the relevant behaviour. A cut-off score of 15 or greater is suggested by the authors of the measure to indicate the presence of autism spectrum disorder. In the current study, the lifetime version of the SCQ was used.

#### *Wessex questionnaire* [32]

This scale quantifies self-help skills for children and adults with intellectual disability, which resulted in its common use in individuals with genetic syndromes [15, 33–35]. The items enquire about a variety of different adaptive skills, forming five subscales: self-help skills, speech, vision, hearing and mobility. For the current study, the self-help total score, with a maximum of 9, was used. Self-help scores of 6 and over are classified as a moderate level of skill. The term ‘typical’ has been used to replace the original questionnaire term ‘normal’, in keeping with current terminology.

### Statistical analysis

Standard principal component analysis (PCA) based on the SCQ items was used to investigate the extent of overlap between the autism profiles across the thirteen genetic groups (Additional file 1: Fig. S1). We conducted PCA to confirm previous findings that indicate PCA, as an unsupervised analysis, is not the right type of analysis to generate autism-related profiles for genetic syndromes [1]. For Additional file 1: Fig. S1, the first two components (PC1 and PC2), which explained the largest amount of the variance, were selected. The PCA revealed two main clusters, separating mostly individuals who use few or more words from individuals who use no words (A. Additional file 1: Fig. S1). Following this, language items were excluded from the analysis resulting in a single cluster (B. Additional file 1: Fig. S1).

The lifetime version of the SCQ was used, and all 40 items were included in the analysis. However, the traditional binary scoring (1 = Yes, 0 = No) was not reflective of the heterogeneity of language ability across the sample (e.g., language delay vs no language use across the lifespan). For this reason, an additional score of 2 was introduced for the six language-related items for all participants to indicate the absence of language use rather than the absence of autistic-related characteristics for these items across the lifespan. This new score allowed us to include all items for all participants in the classification analysis and capture language heterogeneity at the same time. The coding of all items including the language items is processed as categorical (rather than ordinal) by the model. As a result, the model generates a pattern of responses for each syndrome group. A score of 2 is therefore not weighted as more important/influential than a score of 1 or 0 by the model. However, if a group tends to score 2,1 or 0 on most language items, this type of scoring pattern can help create a unique profile for this group.

Similarly to previous studies [29], the SVM approach was adopted to provide better predictive accuracy of genetic groups of the thirteen genetic syndromes based on their SCQ scores. In essence, an SVM training algorithm uses training exemplars (in this case, with each exemplar being the list of item-level SCQ responses for a given individual) with their category classes (in this case syndrome group membership) to build a model which can then be used to classify novel exemplars (cases). To build the model, the SVM maps training exemplars to high-dimensional space in a manner which maximises space between exemplars of different categories, and a hyperplane/set of hyperplanes are constructed to separate the categories.

Technical specifications were also consistent with previously identified optimal parameters (e.g., the use of radial kernel, the choice of cross-validation method and the approach to generating gamma and cost parameters) [29]. The *n*-fold validation uses *n*-1 observations or leave one observation out of the whole sample and builds the SVM classifier on the remaining observations. Previously, this method has allowed for an independent estimate of the accuracy of the entire SVM model on the entire sample [29]. Building the model requires determining the optimal values of the gamma and cost parameters. Using random search of gamma and cost parameter values (up to 100 combinations), the performance of the model was further optimised. Based on the random search, accuracy of the SVM classifier for each combination of gamma and cost parameters was evaluated and the combination of values giving the highest accuracy was chosen. To identify the best parameters, the entire dataset was split in two halves. One half served as the training set, and the other half served as the test set. To deal with unequal sample sizes across syndrome groups, at least fifteen participants from each syndrome group featured in the training set at the stage of identifying the parameters with the highest accuracy. Once the best combination of gama and cost parameters had been identified, the model was trained and tested on the entire sample size, which further helped to reduce over or underestimation of classification accuracy for certain groups.

The final SVM model adopted multiclass classification, reflecting training of multiple binary classifiers, or mapping data points to dimensional space to gain mutual linear separation between every two classes. The multiclass classification uses a ‘one-to-one’, or ‘one-against-one’ approach where k(k-1)/2 (k is the number of the classes) [29]. The final output of the SVM model assigns each of the data points into a ‘predicted’ class, which is the most frequently chosen class by the binary classifiers. Apart from classification accuracy, the decision values of the binary classifiers can also generate predicted probability for each class, which can be an alternative way to assess the confidence of the SVM predictions.

We also carried out an item-level analysis, in which we evaluated each of the SCQ items within each group in order of their importance to the classification results on a scale from 0 to 100, indicating lowest to highest importance, respectively. Although the output of this analysis provides data on all items, we reported the five most and the five least important items for each syndrome group for interpretation purposes and consistent with previous research [29]. For the selection of the items, the following criteria were used: a score of 20 or lower for the five least important items and a score of 50 or higher for the most important items. However, it is important to note that the variability across groups was substantial and some groups evidenced scores as high as 100 for some items (e.g., RTS group), while other groups evidenced scores of 50 for most items (e.g., FXS group). This indicates that for some groups, most SCQ items might have been equally important for their classification results, while for others only certain items might have been particularly important. The selection of the cut-off criteria was data-driven and exploratory. In line with previous work [29], this study adopted libSVM, via the SVM function in the e1071 library in R [36, 37].

## Results

### SVM classification

**Fig. 1:**
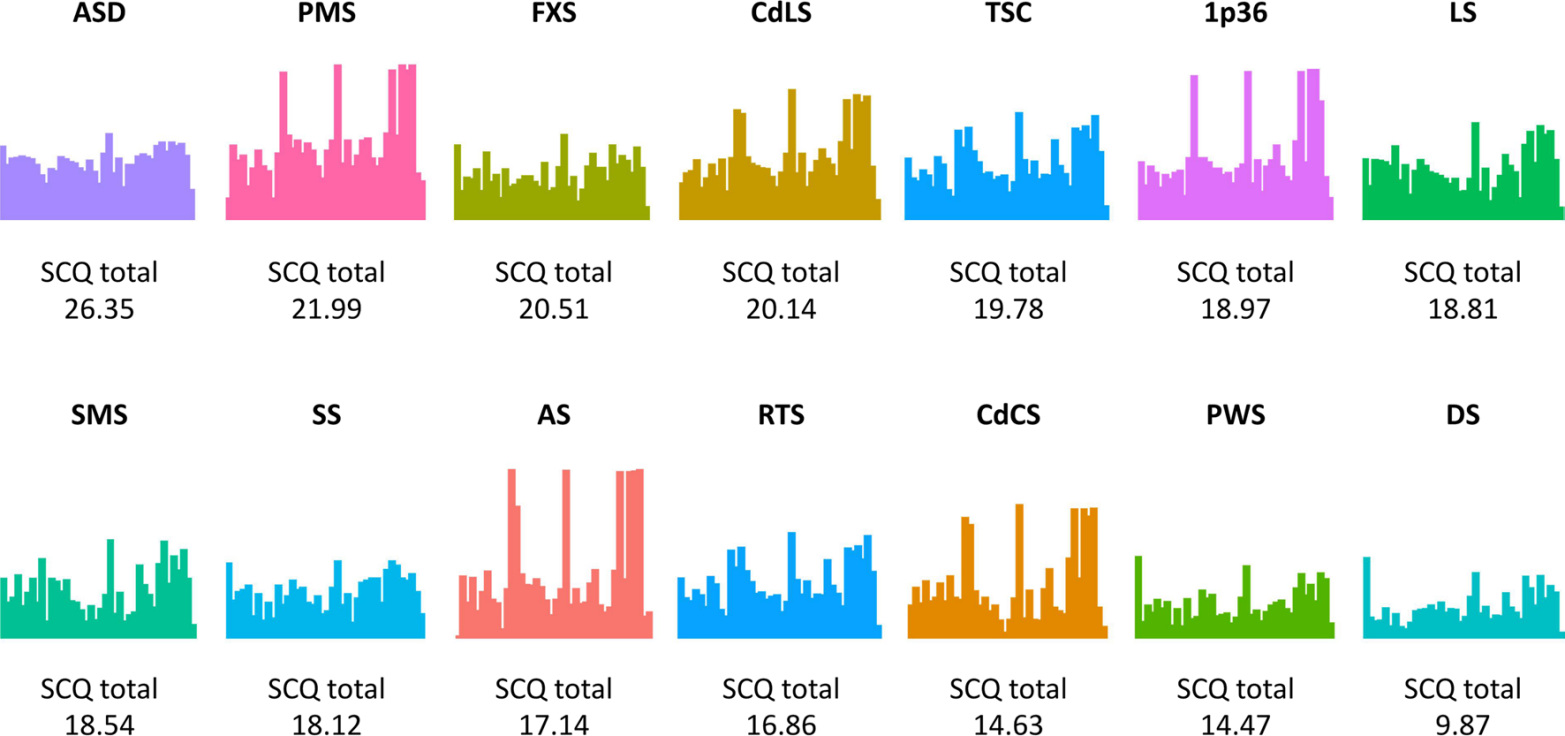
Visual representation of the SCQ profiles in individuals with genetic syndromes and autistic individuals. This figure provides a visual representation of the diversity and similarity of SCQ profiles across genetic syndromes and their distinctiveness compared to the SCQ profile of the autistic group with genetic syndromes. The x axis represents each of the 40 SCQ items. The y axis represents the overall pattern of responses (i.e., “Yes (1)”/”No (0)”/”No language use (2)”) for each item for the respective genetic group. The different colours allow discrimination between the groups. *AS* Angelman syndrome, *FXS* Fragile X syndrome, *PWS* Prader–Willi syndrome, *RTS* Rubinstein–Taybi syndrome, *CdLS* Cornelia de Lange syndrome, *DS* Down syndrome, *CdCS* Cri du Chat syndrome, *LS* Lowe syndrome, *SMS* Smith–Magenis syndrome, *SS* Sotos syndrome, *TSC* Tuberous sclerosis complex, *PMS* Phelan-McDermid syndrome, *1p36* 1p6 deletion syndrome, *ASD* Autism spectrum disorder. *Total* All individuals with genetic syndromes, *SCQ* Social Communication Questionnaire

To address Aim 1, advanced statistical methods, which allowed inclusion of the genotype membership into the analysis, were adopted. Using this multiclass approach, 55% of the individuals with genetic syndromes were accurately classified in the appropriate genetic group (Table 2 and Fig. 1). In comparison, analysis such as PCA does not integrate knowledge of the genotype or classification approaches. Instead, they provide a good representation of the overlap between different individuals across the different groups, which, however, did not allow the opportunity to generate autism-related profiles based on the SCQ scores (Additional file 1: Fig. S1). Groups with the highest classification accuracy (AS, FXS, PWS, RTS) and moderate classification accuracy (CdCS, CdLS, 1p36, SMS) showed the highest post hoc predicted probability for allocation to the appropriate group. By contrast, groups with the lowest classification accuracy (LS, SS, TSC, PMS) showed equal post hoc predicted probability for groups different from their group (Table 2).

**Table 2:** SVM results: SVM frequency of assigned group/class and predicted probabilities. Groups presented in order of classification accuracy

Genetic syndromes																											
Genetic syndrome		AS		FXS		PWS		RTS		CdLS		DS		CdCS		1p36		LS		SMS		TSC		SS		PMS	Total
	*n*	Prob	*n*	Prob	*n*	Prob	*n*	Prob	*n*	Prob	*n*	Prob	*n*	Prob	*n*	Prob	*n*	Prob	*n*	Prob	*n*	Prob	*n*	Prob	*n*	Prob	*n*
AS	**130**	**0.48**	7	0.02	6	0.02	1	0.02	28	0.10	6	0.03	15	0.14	5	0.08	9	0.07	5	0.06	3	0.04	8	0.10	1	0.02	224
FXS	4	0.06	**237**	**0.42**	41	0.13	7	0.05	30	0.13	7	0.10	14	0.11	9	0.10	38	0.21	25	0.16	27	0.17	5	0.12	18	0.26	462
PWS	1	0.04	35	0.14	**219**	**0.41**	8	0.07	39	0.11	60	0.29	9	0.09	3	0.08	15	0.14	9	0.12	26	0.16	5	0.08	15	0.21	444
RTS	0	0.01	1	0.03	0	0.02	**78**	**0.63**	0	0.02	0	0.02	0	0.01	0	0.02	0	0.02	1	0.02	0	0.02	0	0.02	0	0.03	80
CdLS	12	0.12	12	0.09	3	0.08	7	0.05	**97**	**0.30**	5	0.08	7	0.12	13	0.13	15	0.13	8	0.13	19	0.15	15	0.14	3	0.10	216
DS	1	0.02	5	0.06	9	0.12	1	0.03	3	0.06	**55**	**0.29**	2	0.05	2	0.06	5	0.07	0	0.04	2	0.08	1	0.06	3	0.10	89
CdCS	5	0.07	0	0.02	0	0.02	0	0.01	2	0.04	2	0.02	**28**	**0.29**	1	0.05	1	0.03	2	0.05	0	0.02	0	0.03	0	0.02	41
1p36	0	0.03	0	0.02	0	0.01	0	0.02	0	0.03	0	0.02	0	0.02	**8**	**0.20**	0	0.03	0	0.02	0	0.04	0	0.08	0	0.02	8
LS	0	0.04	0	0.07	0	0.05	0	0.03	0	0.06	0	0.04	0	0.05	0	0.08	**6**	**0.14**	0	0.08	0	0.07	0	0.06	0	0.05	6
SMS	0	0.02	0	0.04	0	0.03	0	0.02	0	0.04	0	0.02	0	0.03	0	0.05	0	0.04	**4**	**0.20**	0	0.03	0	0.03	0	0.03	4
TSC	1	0.04	0	0.05	0	0.05	0	0.03	0	0.06	0	0.05	0	0.04	0	0.07	0	0.06	0	0.05	**6**	**0.16**	0	0.09	0	0.07	7
SS	0	0.01	0	0.03	0	0.03	0	0.02	0	0.03	0	0.03	0	0.02	0	0.03	0	0.03	0	0.03	0	0.04	**1**	**0.15**	0	0.02	0
PMS	0	0.04	0	0.02	0	0.01	0	0.02	0	0.03	0	0.01	0	0.02	0	0.08	0	0.02	0	0.02	0	0.04	0	0.03	**0**	**0.06**	1
Total	154		297		278		102		199		135		75		41		89		54		83		35		40		1582
Accuracy (%)	130/154 (84%)		237/297 (80%)		219/278 (79%)		78/102 (77%)		97/199 (49%)		55/135 (41%)		28/75 (37%)		8/41 (20%)		6/89 (7%)		4/54 (7%)		6/83 (7%)		1/35 (0.3%)		0/40 (0%)		868/1582 (55%)

*AS* Angelman syndrome, *CdCS* Cri du Chat syndrome, *1p36* 1p6 deletion syndrome, *CdLS* Cornelia de Lange syndrome, *FXS* Fragile X syndrome, *PWS* Prader–Willi syndrome, *LS* Lowe syndrome, *SMS* Smith–Magenis syndrome, *DS* Down syndrome, *SS* Sotos syndrome, *RTS* Rubinstein–Taybi syndrome, *TSC* Tuberous sclerosis complex, *PMS* Phelan-McDermid syndrome, *Prob* Predicted Probability, *N* Sample size

Bold in each row correspond to the predicted probability and the number of individuals assigned to the syndrome group for this particular group

The validity of the prediction model was also tested by correlating the predicted probabilities with the number of individuals correctly assigned to the relevant genetic class/group. As previously shown [29], strong, positive correlations between the predicted probability and the number of individuals correctly assigned to the relevant genetic group emerged (see Additional file 1: Table S1), which highlights the validity of the prediction model.

After including all participants in the initial analysis, a sensitivity analysis was also conducted with the participants who met or scored above the suggested cut-off scores for ASD on the SCQ. Both sets of analyses generated comparable results, suggesting that variation in SCQ scores (i.e., higher scores) did not influence the generation of the identified profiles.

### Misallocation

The poor prediction for some of the groups was a result of frequent misallocation to a specific other genetic syndrome group. For instance, individuals from the CdCS and CdLS groups were most frequently misclassified into the AS group; individuals from the LS, SMS, SS into the FXS group; individuals from the DS and the RTS into the PWS group; individuals from the 1p36 and PMS into the CdLS group and individuals from the TSC group were equally likely to be misclassified into the CdLS, FXS and PWS groups.

### Self-help skills as an additional predictor

To address Aim 2, the analysis was repeated adding self-help scores as an additional predictor. The average accuracy of the SVM model remained the same (55%), suggesting that self-help skills were not a confounding factor. However, the prediction accuracy for some of the genetic groups slightly improved (CdCS, PWS, DS, TSC, PMS 1p36) or declined (AS, CdLS, RTS), suggesting that self-help skills might explain a small proportion of the variance in autistic characteristics for these groups (between 2 and 9%) (Additional file 1: Table S2). For example, AS and PMS were both associated with lower self-help scores, but autism-related profiles were clearly dissociable and classification accuracy differed, suggesting that the manifestation of autistic characteristics is at least partly independent of self-help skills in these groups.

### Inclusion of the autistic group

To understand the effect of non-syndromic autism on the classification accuracy, a group of autistic individuals without a genetic syndrome was included in the analysis (Aim 3). It was predicted that syndrome groups showing high levels of autistic characteristics and low classification accuracy will be more likely to be misclassified into the autistic group. The addition of the autistic group did not change the average accuracy of the model. However, the classification accuracy of the FXS group reduced substantially. Individuals with FXS were more likely to be misclassified into the autistic group and vice versa (Additional file 1: Table S3), despite high classification accuracy for both groups, suggesting that the FXS and the autistic groups show specific but overlapping phenotypes. To a lesser extent, individuals in the PWS and the TSC groups were also misclassified into the FXS or/and the autistic group. Regarding AS, RTS, CdLS, CdCS, DS, 1p36, LS, SMS, and PMS, they remained more likely to be misclassified as other syndrome groups, regardless of small improvements in classification accuracy, suggesting that lower phenotypic specificity does not necessarily reflect a greater phenotypic overlap with the autistic group for all individuals or groups (Additional file 1: Table S3).

### Item-level analysis

**Fig. 2:**
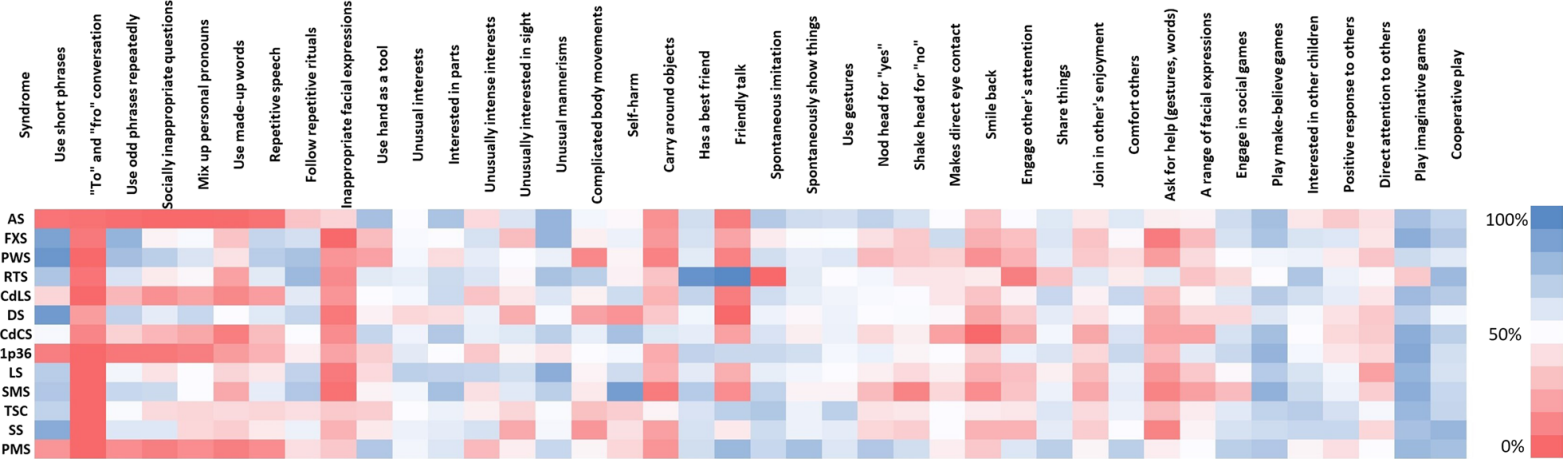
Heatmap – distribution of SCQ items for each syndrome group. “Blue” indicates that more than 50% of the individuals in this group scored “Yes (1)” on the respective item. “White/No colour” indicates that ~ 50% of the individuals scored “Yes (1)” on the respective item. “Red” indicates that less than 50% of the individuals scored “Yes (1)”. The saturation of the colour indicates the number of individuals (the darkest blue indicates all individuals scored “Yes (1)”, and the darkest red indicates none of the participants scored “Yes (1)” on this item. (*Notes* The non-verbal participants were coded as non-responders for the items requiring a verbal ability for the purposes of this figure). *AS* Angelman syndrome, *FXS* Fragile X syndrome, *PWS* Prader–Willi syndrome, *RTS* Rubinstein–Taybi syndrome, *CdLS* Cornelia de Lange syndrome, *DS* Down syndrome, *CdCS* Cri du Chat syndrome, *LS* Lowe syndrome, *SMS* Smith–Magenis syndrome, *SS* Sotos syndrome, *TSC* Tuberous sclerosis complex, *PMS* Phelan-McDermid syndrome, *1p36* 1p6 deletion syndrome

To address Aim 4, a within-syndrome analysis revealed that the top five most and least contributing items varied as a function of group (Table 3 and Fig. 2). Items enquiring about the quality of social interaction (item 20), imaginative play (item 32) and complicated body movements/gestures (items 16) were identified within the top five items contributing to classification accuracy across a large proportion of syndromes (e.g., FXS, PWS, RTS, 1p36, LS, SMS, TSC, PMS). By contrast, items related to communication (Items 2, 6, 7, 3, 4) contributed substantially to the classification prediction of a smaller proportion of the syndrome groups (RTS, 1p36, DS, TSC). A mixture of the listed items contributed to the prediction accuracy of the syndrome groups with both the highest (AS) and the lowest (PMS) classification accuracy (Fig. 1). For some syndrome groups (e.g., AS, CdLS, CdCS), most items had equal contributions to the model. Crucially, lower contribution of certain items does not suggest an absence of the particular autistic characteristic for the particular group. Instead, lower contribution indicates that this item does not contribute considerably to the performance of the model.

**Table 3:** The level of contribution of each SCQ item to the predictions of the SVM model

SCQ characteristics	AS	FXS	PWS	RTS	CdLS	DS	CdCS	1p36	LS	SMS	TSC	SS	PMS	ASD
1. Short phrases						−		+						
2. Reciprocal Conversation				+		+		+						
3. Odd phrases	+			+		+					+			
4. Inappropriate questions		−		+	−	+				−	+	−		−
5. Pronoun reversal				+	−	+		+	−	−	+		+	
6. Made up words			−	+		+	−				+			
7. Phrase/word repetition											+			
8. Repetitive rituals									−	−				
9. Inappropriate facial expressions		−									−		−	
10. Use others as tools			−	−									−	−
11. Unusual interests			−					−					−	
12. Interested in parts of objects			−	−				−			−		−	−
13. Unusually intense interests					−		−						−	
14. Unusual interest in smell, taste	+								−			+		
15. Odd mannerisms														
16. Complicated body movements	+	+	+		+		+		+	+		+	+	+
17. Self−harm			+											
18. Carry objects around		−			−			−	−		−			
19. Best friend	+		+		+		+							+
20. Friendly conversation		+			+		+	+	+	+		+	+	+
21. Spontaneous imitation						−								
22. Spontaneously show interest	−	+		−	−						−	−		−
23. Use gestures						−								
24. Nod head for "yes"	−						−							
25. Shaked head for "no"		−												
26. Face-to-face conversation														
27. Smile back	−			−			−				−			−
28. Special interests														
29. Share things with others	−									+				
30. Invite to join in their enjoyment										−		−		
31. Offer comfort		−				−								
32. Eye contact		+	+		+		+	+	+	+		+	+	+
33. A range of facial expressions						−		−						
34. Spontaneous social play														
35. Pretend games														
36. Interested in unknown peers										−		−		
37. Positive approach to peers														
38. Pay attention to one’s voice	−		−	−			−							
39. Imaginative play	+	+	+		+		+		+	+		+	+	+
40. Cooperative play								−	+			−		

+ reflects the top five items, which contribute the most to the model,—represents the top 5 items, which contribute the least to the model for each syndrome group. These symbols ( ±) do not represent high or low scores on these items, they represent the level of contribution to the classification model

*AS* Angelman syndrome, *CdCS* Cri du Chat syndrome, *1p36* 1p6 deletion syndrome, *CdLS* Cornelia de Lange syndrome, *FXS* fragile X syndrome, *PWS* Prader–Willi syndrome, *LS* Lowe syndrome, *SMS* Smith–Magenis syndrome, *DS* Down syndrome, *SS* Sotos syndrome, *RTS* Rubinstein–Taybi syndrome, *TSC* Tuberous sclerosis complex, *PMS* Phelan-McDermid syndrome, *ASD* Autism spectrum disorder

## Discussion

This study extended previous findings of different but partially overlapping autism profiles across thirteen genetic syndromes [29] in a large sample of 1582 individuals. This approach allowed us to gain a detailed understanding of syndrome-associated profiles of autistic characteristics across a large number of syndrome groups, using a consistent screening tool. The findings highlight the need to refine measures of autism for use in this population, in order to improve the precision of assessment of autism and related needs in these groups. This will be beneficial to ensure timely and effective access to the most appropriate support which takes into consideration these syndrome-associated autism profiles.

The current study indicated substantial overall accuracy of the model (55%). While findings indicated a lower number of correctly classified individuals (i.e., classification accuracy) (55%) compared to earlier work (63%) [29], the inclusion of such a large number of genetic syndrome groups is likely to have increased the chance of misclassification relative to previous work; as SVM is originally designed for binary rather than multiclass/group classification. Nevertheless, the classification accuracy of the model is substantial given the inclusion of 13 groups. This large and diverse sample further enables greater understanding of whether variation in sample size affects misclassification patterns for individual syndromes. For instance, classification accuracy for the DS group was higher in this study with a larger sample of individuals with DS (*n* = 135, 41%) compared to previous classification reports (*n* = 21, 10%). However, individuals from the DS group remained to be most misclassified into the PWS group across both studies [29], indicating consistent profile similarities and differences across syndromes regardless of the sample size. Larger sample sizes may therefore improve classification accuracy as a function of mathematical dependency (i.e., the more individuals in a group, the more opportunities of accurate classification). However, the misclassification patterns (i.e., the *type* of groups the misclassified individuals are placed in) do not vary as a function of sample size. Furthermore, the CdLS group (*n* = 199) was of a comparable size to the AS group (*n* = 154), and still showed considerably lower classification accuracy (49%) compared to the AS group (84%), indicating lower phenotypic specificity of autistic traits in the CdLS group compared to the AS group irrespective of sample size. Nevertheless, variable sample sizes might have resulted in lower accuracy for smaller groups (*n* = 40 or less), which reduces the generalisability of our results. Future studies should consider either using groups with identical sample size (i.e., minimum *n* = 200 for each group) or a very large total sample size (*n* = 6000 or more participants) to confirm these findings. Given the rarity of some syndrome groups, a combined effort from multiple research centres across the world is likely to increase the chance of providing conclusive evidence.

In line with the accuracy scores and based on visual inspection of the data (Fig. 1), it was further observed that four of the genetic syndromes (AS, FXS, PWS, RTS) were associated with distinct autistic profiles, which were clearly separable from the profiles of other groups (CdLS, DS, CdCS, 1p36, LS, SMS, TSC, SS and PMS) (70% or more classification accuracy). By contrast, the rest of the syndrome groups either showed partial (e.g., CdCS, CdLS, DS, 1p36 deletion), or no profile separability (e.g., LS, SS, SMS, TSC, PMS) (7% or less classification accuracy). These findings are consistent with earlier autism-related phenotyping in genetic syndromes using parametric comparisons [15]. Nevertheless, a noticeable overlap is also observed across syndrome groups with relatively distinct autism profiles (e.g., pronounced social interaction difficulties or differences in CdLS and FXS) [27, 38–41]. Together, these findings suggest a considerable autism-related phenotypic overlap between different syndrome groups and considerable autism-related phenotypic variability within syndrome groups, supporting recent findings in genetic syndromes [25]. In line with the studies, the findings provide further evidence that the presence of a genetic syndrome seems to increase the likelihood of both autistic traits and autism profiles that are subtly different in nature relative to that observed in autistic individuals who do not have a genetic syndrome. The findings also highlight the importance of considering both within- and between-syndrome similarities and differences as part of clinical and educational support services for individuals with genetic syndromes who show autistic characteristics.

Documenting autistic characteristics in individuals with varying levels of intellectual functioning is particularly challenging [21].While it was not possible to evaluate the effect of cognitive impairment specifically in this study, we were able to consider the contribution of self-help skills (as an estimate of intellectual functioning). In the current analysis, self-help skills were added as an additional predictor. Although the addition did not change the average classification accuracy, some groups showed minimal improvement (CdCS, PWS, DS, TSC, PMS, 1p36) or decrease (AS, CdLS, RTS) in accuracy. This finding indicates that self-help skills might explain a small proportion of the variance in autistic characteristics, although the manifestation of autistic characteristics across syndrome groups appears largely independent of self-help skills, supporting previous findings [20, 29, 42, 43]. Future research should still consider investigating whether the degree of intellectual functioning, language ability and self-help skills affects the diagnostic process and access to support. For instance, individuals with genetic syndromes and higher scores on self-help skills measures might have an atypical presentation of autistic traits or engage in compensatory strategies, resulting in further diagnostic uncertainty and overshadowing. Diagnostic uncertainty is then expected to result in delayed or limited access to support. Delayed support could be particularly damaging for this cohort of individuals, as they often experience a high degree of autism-related challenges despite the heterogeneity of observable autistic traits [21].

The inclusion of individuals who speak no or few words resulted in two clearly separable PCA clusters and higher attribution of importance to items measuring atypical communication by the model (Additional file 1: Fig. S1). A single PCA cluster and higher attribution to items assessing quality of social interaction (i.e., cooperative play) emerged only after the exclusion of the communication-related items, consistent with earlier studies including individuals who speak in full sentences [29]. This finding suggests that quality of social interactions might capture the specificity of autism profiles across syndrome groups who speak few or more words. By contrast, broad communication difficulties or differences might be more characteristic for syndrome groups, who use/speak no words regardless of whether they meet diagnostic cut-off for autism. These findings suggest that capturing variability in social-communicative skills across syndrome groups might improve diagnostic sensitivity and specificity in genetic syndromes. In particular, individuals who have few or no words might still have a pronounced need for autism-related support, but differential diagnosis of autism in these individuals using traditional autism diagnostic assessment tools is likely to be challenging. Future research may consider developing observational measures, which rely less on linguistic ability to assess the diagnostic utility of social-communicative characteristics. Observational measures of autistic characteristics, for instance, have shown to capture variability in phenotypic expression of autistic characteristics in genetic syndromes better than parent-report measures [44, 45].

The identification of similarities and differences in autism profiles across autistic individuals with and without genetic syndromes has important clinical implications. A high degree of overlap between profiles would suggest that existing gold standard autism assessments would be equally suitable for individuals with genetic syndromes, whereas substantial variability across profiles might hinder the validity of such assessments, which were not designed with such populations and associated variability in mind. For this reason, a group of autistic individuals without a genetic syndrome was also added to the current model, resulting in substantial misclassification of individuals with FXS into the autism group and vice versa. This finding supports previous evidence of substantial phenotypic overlap [46] between individuals with FXS and autistic individuals and further suggests that a traditional approach to autism diagnosis and treatment could be beneficial to individuals with FXS. To a lesser degree, the TSC and PWS showed a similar pattern to the autistic individuals, supporting previously identified social-communicative difficulties or differences as key phenotypic similarities between autism and FXS [42, 43] autism and TSC [47] and autism and PWS [48, 49]. These findings indicate that greater precision of assessment may be necessary in these populations, and this requires more in depth/fine-grained evaluation and less reliance of traditional, broad brush, cut-off scores. A likely explanation is that traditional autism assessments rarely factor in the presence of varying levels of language development and intellectual disability [54]. Consequently, syndrome groups with a greater incidence of typical speech/language development or higher adaptive skills scores have more opportunities to score on the SCQ measure compared to syndrome groups with a lower incidence. Future research should thus focus on evaluating whether diagnostic assessments adjusted to the individual profile of autistic characteristics identified in a given genetic syndrome will lead to higher diagnostic certainty and timely provision of support for autistic individuals with genetic syndromes compared to traditional assessments. Item-level analysis conducted in this study provides a basis for refinement of assessment tools to improve sensitivity and specificity for detecting syndrome-associated autism profiles. This analysis clearly demonstrated that a different selection of items has contributed to the model differentially across groups, suggesting that the development of precise and personalised assessments would require a differential selection of diagnostic items across syndrome groups, as well. In line with earlier propositions, the presence of a genetic syndrome seems to increase the propensity for autistic characteristics and syndrome-associated profiles, but additional factors might be necessary to meet current diagnostic cut-off for autism [50–53].

### Limitations

The SCQ [30] focuses primarily on social interaction and communication abilities, with a less pronounced focus on repetitive and restricted behaviours, during a specific developmental period (4–5 years). On the one hand, this specific developmental period allows a comparison across individuals with a wide age range. On the other hand, divergent developmental pathways for autistic characteristics have already been observed in genetic syndromes (FXS and CdLS, CdLS and CdCS), which have been defined by pronounced phenotypic similarities in early development [54]. Cross-sectional studies should therefore be interpreted cautiously as the manifestation/level of autistic characteristics across genetic syndromes, and heterogeneous autism seems to vary as a function of age. Although parents and carers were asked to consider their children’s behaviour across the lifespan, the parents and carers of older participants might have been more likely to experience a reporting bias for the questions concerning their child’s behaviours between 4 and 5 years of age, further indicating the low specificity of the SCQ for older individuals. Additionally, the presence of an ASD diagnosis in individuals with genetic syndromes and the type of professional, who diagnosed autistic individuals with ASD, were not always provided by the parents/carers. Different professionals also provided an ASD diagnosis for the autistic individuals without a known genetic syndromes and some of them (e.g., General Practitioners) might not have received a specialist ASD training, which might affect the reliability of the diagnosis. Future research should aim to address the imbalance in sample size across groups by including syndrome groups with a similar sample size and as many individuals in each syndrome group as possible. Future research should also consider adopting a longitudinal approach and developing syndrome-associated observational measures which could capture variability in phenotypic expression across the lifespan better than one-time parent-report measures [44]. Furthermore, the SCQ is primarily a screening tool used to evaluate possible autistic characteristics and therefore cannot be relied upon to provide an extensive assessment of autism. Future studies should therefore consider replication of the study findings using more in depth autism assessment tools. Additionally, visualising and making meaningful inferences about the nature of the hyperplanes used by SVM is challenging due to the “black box” nature of the model. Future studies should therefore compare different machine learning algorithms/paradigms to determine which best distinguish autistic profiles across different genetic syndromes and consider stratifying the analyses by taking into account verbal ability/diagnosis status/professional providing the diagnosis. The use of considerably larger group sizes will allow the implementation of this type of analysis without losing power.

## Conclusions

Using supervised machine learning, this study confirms the presence of different but overlapping autism profiles across the thirteen genetic syndromes in approximately 1500 individuals. Defining aspects of phenotypic variability and similarity across different genetic groups has the potential to improve the accuracy of autism identification in individuals with genetic syndromes associated with intellectual disability. The findings highlight the need for consideration of syndrome-associated profiles of autism in order to improve the precision of autism screening, diagnostic assessment and support services for individuals in these populations [55]. Although this study focuses specifically on autistic characteristics in individuals with genetic syndromes, a syndrome-associated clinical evaluation for all aspects of each syndrome is equally important and would require the same personalised approach.

## Supplementary Information

**Additional file 1: Fig. S1.** PCA plots of SCQ-generated autism profiles in the thirteen genetic syndromes. **Table S1.** Correlations between the number of individuals assigned to each group and the post-hoc predicted probability. **Table S2.** SVM results after the addition of self-help skills (WESSEX) as an additional predictor. **Table S3.** SVM results after the addition of the ASD group

## Data Availability

The datasets used and/or analysed during the current study are available from the corresponding author on reasonable request.

## References

[1] Tick B, Bolton P, Happé F, Rutter M, Rijsdijk F (2016). Heritability of autism spectrum disorders: a meta-analysis of twin studies. J Child Psychol Psychiatry.

[2] Association AP. Diagnostic and statistical manual of mental disorders (DSM-5®): American Psychiatric Pub; 2013. 10.1590/s2317-17822013000200017 24413388

[3] Happé F, Ronald A (2008). The ‘fractionable autism triad’: a review of evidence from behavioural, genetic, cognitive and neural research. Neuropsychol Rev.

[4] Havdahl A, Niarchou M, Starnawska A, Uddin M, van der Merwe C, Warrier V (2021). Genetic contributions to autism spectrum disorder. Psychol Med..

[5] Ronald A, Happé F, Plomin R (2005). The genetic relationship between individual differences in social and nonsocial behaviours characteristic of autism. Dev Sci.

[6] Ronald A, Happé F, Bolton P, Butcher LM, Price TS, Wheelwright S (2006). Genetic heterogeneity between the three components of the autism spectrum: a twin study. J Am Acad Child Adolesc Psychiatry.

[7] Dworzynski K, Happé F, Bolton P, Ronald A (2009). Relationship between symptom domains in autism spectrum disorders: a population based twin study. J Autism Dev Disord.

[8] Warrier V, Toro R, Won H, Leblond CS, Cliquet F, Delorme R (2019). Social and non-social autism symptoms and trait domains are genetically dissociable. Commun Biol.

[9] Happe F (2006). Understanding autism: from basic neuroscience to treatment. Nature.

[10] Robinson EB, Koenen KC, McCormick MC, Munir K, Hallett V, Happé F (2012). A multivariate twin study of autistic traits in 12-year-olds: testing the fractionable autism triad hypothesis. Behav Genet.

[11] Ronald A, Larsson H, Anckarsäter H, Lichtenstein P (2011). A twin study of autism symptoms in Sweden. Mol Psychiatry.

[12] Gotts SJ, Simmons WK, Milbury LA, Wallace GL, Cox RW, Martin A (2012). Fractionation of social brain circuits in autism spectrum disorders. Brain.

[13] Muhle R, Trentacoste SV, Rapin I (2004). The genetics of autism. Pediatrics.

[14] Persico AM, Bourgeron T (2006). Searching for ways out of the autism maze: genetic, epigenetic and environmental clues. Trends Neurosci.

[15] Oliver C, Berg K, Moss J, Arron K, Burbidge C (2011). Delineation of behavioral phenotypes in genetic syndromes: characteristics of autism spectrum disorder, affect and hyperactivity. J Autism Dev Disord.

[16] Richards C, Jones C, Groves L, Moss J, Oliver C (2015). Prevalence of autism spectrum disorder phenomenology in genetic disorders: a systematic review and meta-analysis. The Lancet Psychiatry.

[17] Dunn K, Rydzewska E, Henderson A, Cooper S-A (2016). The health of Scotland’s 5,709 people with autism and intellectual disabilities. J Intellect Disabil Res.

[18] Cornish K, Scerif G, Karmiloff-Smith A (2007). Tracing syndrome-specific trajectories of attention across the lifespan. Cortex.

[19] Hall SS, Lightbody AA, Hirt M, Rezvani A, Reiss AL (2010). Autism in fragile X syndrome: A category mistake?. J Am Acad Child Adolesc Psychiatry.

[20] Mount RH, Charman T, Hastings RP, Reilly S, Cass H (2003). Features of autism in Rett syndrome and severe mental retardation. J Autism Dev Disord.

[21] Moss J, Howlin P (2009). The assessment and presentation of autism spectrum disorders in genetic syndromes: implications for diagnosis, intervention and understanding the wider ASD population. J Intellect Disabil Res.

[22] Moss J, Howlin P, Magiati I, Oliver C (2012). Characteristics of autism spectrum disorder in Cornelia de Lange syndrome. J Child Psychol Psychiatry.

[23] Trillingsgaard A, Østergaard JR (2004). Autism in Angelman syndrome: an exploration of comorbidity. Autism.

[24] Lee NR, Niu X, Zhang F, Clasen LS, Kozel BA, Smith A (2022). Variegation of autism related traits across seven neurogenetic disorders. Transl Psychiatry.

[25] Chawner SJ, Doherty JL, Anney RJ, Antshel KM, Bearden CE, Bernier R (2021). A genetics-first approach to dissecting the heterogeneity of autism: phenotypic comparison of autism risk copy number variants. Am J Psychiatry.

[26] Oliver C, Hagerman R. Trends and challenges in behavioural phenotype research. 2007.

[27] Moss JF, Oliver C, Berg K, Kaur G, Jephcott L, Cornish K (2008). Prevalence of autism spectrum phenomenology in Cornelia de Lange and Cri du Chat syndromes. Am J Ment Retard.

[28] Sloneem J, Moss J, Powell S, Hawkins C, Fosi T, Richardson H (2022). The prevalence and profile of autism in Sturge-Weber syndrome. J Autism Dev Disord.

[29] Bruining H, Eijkemans MJ, Kas MJ, Curran SR, Vorstman JA, Bolton PF (2014). Behavioral signatures related to genetic disorders in autism. Mol Autism.

[30] Rutter M, Bailey A, Lord C. SCQ. The Social Communication Questionnaire Torrance, CA: Western Psychological Services; 2003.

[31] Rutter M, Le Couteur A, Lord C. ADI-R. Autism diagnostic interview revised Manual Los Angeles: Western Psychological Services; 2003.

[32] Kushlick A, Blunden R, Cox G (1973). A method of rating behaviour characteristies for use in large scale surveys of mental handicap. Psychol Med.

[33] Wilde L, Wade K, Eden K, Moss J, de Vries P, Oliver C (2018). Persistence of self-injury, aggression and property destruction in children and adults with tuberous sclerosis complex. J Intellect Disabil Res.

[34] Moss J, Oliver C, Arron K, Burbidge C, Berg K (2009). The prevalence and phenomenology of repetitive behavior in genetic syndromes. J Autism Dev Disord.

[35] Skuse DH, James R, Bishop DV, Coppin B, Dalton P, Aamodt-Leeper G (1997). Evidence from Turner's syndrome of an imprinted X-linked locus affecting cognitive function. Nature.

[36] Meyer D, Wien FT. Support vector machines. Interface Libsvm Package e1071. 2015.

[37] Chang C-C, Lin C-J (2011). LIBSVM: a library for support vector machines. ACM Trans Intell Syst Technol (TIST).

[38] Moss J, Howlin P, Hastings RP, Beaumont S, Griffith GM, Petty J (2013). Social behavior and characteristics of autism spectrum disorder in Angelman, Cornelia de Lange, and Cri du Chat syndromes. Am J Intellect Dev Disabil.

[39] Moss J, Oliver C, Nelson L, Richards C, Hall S (2013). Delineating the profile of autism spectrum disorder characteristics in Cornelia de Lange and fragile X syndromes. Am J Intellect Dev Disabil.

[40] Moss J, Nelson L, Powis L, Waite J, Richards C, Oliver C (2016). A comparative study of sociability in Angelman, Cornelia de Lange, Fragile X, Down and Rubinstein Taybi syndromes and autism spectrum disorder. Am J Intellect Dev Disabil.

[41] Clifford S, Dissanayake C, Bui QM, Huggins R, Taylor AK, Loesch DZ (2007). Autism spectrum phenotype in males and females with fragile X full mutation and premutation. J Autism Dev Disord.

[42] Klusek J, Martin GE, Losh M (2014). A comparison of pragmatic language in boys with autism and fragile X syndrome. J Speech Lang Hear Res.

[43] Smith LE, Barker ET, Seltzer MM, Abbeduto L, Greenberg JS (2012). Behavioral phenotype of fragile X syndrome in adolescence and adulthood. Am J Intellect Dev Disabil.

[44] Harris SW, Hessl D, Goodlin-Jones B, Ferranti J, Bacalman S, Barbato I (2008). Autism profiles of males with fragile X syndrome. Am J Ment Retard.

[45] Capal JK, Williams ME, Pearson DA, Kissinger R, Horn PS, Murray D (2021). Profile of autism spectrum disorder in tuberous sclerosis complex: results from a longitudinal, prospective, multisite study. Ann Neurol.

[46] Lee M, Martin GE, Berry-Kravis E, Losh M (2016). A developmental, longitudinal investigation of autism phenotypic profiles in fragile X syndrome. J Neurodev Disord.

[47] Jeste SS, Varcin KJ, Hellemann GS, Gulsrud AC, Bhatt R, Kasari C (2016). Symptom profiles of autism spectrum disorder in tuberous sclerosis complex. Neurology.

[48] Dimitropoulos A, Ho A, Feldman B (2013). Social responsiveness and competence in Prader–Willi syndrome: direct comparison to autism spectrum disorder. J Autism Dev Disord.

[49] Zyga O, Russ S, Ievers-Landis CE, Dimitropoulos A (2015). Assessment of pretend play in Prader–Willi syndrome: a direct comparison to autism spectrum disorder. J Autism Dev Disord.

[50] Geschwind DH (2011). Genetics of autism spectrum disorders. Trends Cogn Sci.

[51] Woodbury-Smith M, Scherer SW (2018). Progress in the genetics of autism spectrum disorder. Dev Med Child Neurol.

[52] Bai D, Yip BHK, Windham GC, Sourander A, Francis R, Yoffe R (2019). Association of genetic and environmental factors with autism in a 5-country cohort. JAMA Psychiat.

[53] Karimi P, Kamali E, Mousavi SM, Karahmadi M (2017). Environmental factors influencing the risk of autism. J Res Med Sci Offi J Isfahan Univ Med Sci.

[54] Cochran L, Moss J, Nelson L, Oliver C, editors. Contrasting age related changes in autism spectrum disorder phenomenology in Cornelia de Lange, fragile X, and Cri du Chat syndromes: results from a 2.5 year follow‐up. American Journal of Medical Genetics Part C: Seminars in Medical Genetics; 2015: Wiley Online Library. 10.1002/ajmg.c.31438 25989416

[55] Insel TR (2014). The NIMH research domain criteria (RDoC) project: precision medicine for psychiatry. Am J Psychiatry.

