# Supplementary material for: Profiles of autism characteristics in thirteen genetic syndromes: a machine learning approach
Source: Mol Autism. 2023 Jan 13;14:3. doi: 10.1186/s13229-022-00530-5 (PMC9837969; doi:10.1186/s13229-022-00530-5)
Supplement: Supplementary file 1 — Additional file 1: Fig. S1. PCA plots of SCQ-generated autism profiles in the thirteen genetic syndromes. Table S1. Correlations between the number of individuals assigned to each group and the post-hoc predicted probability. Table S2. SVM results after the addition of self-help skills (WESSEX) as an additional predictor. Table S3. SVM results after the addition of the ASD group [file 13229_2022_530_MOESM1_ESM.docx]

**Supplementary Material**

**PCA Analysis**

**Supplementary Figure 1**. PCA plots of SCQ-generated autism profiles in the thirteen genetic syndromes

**
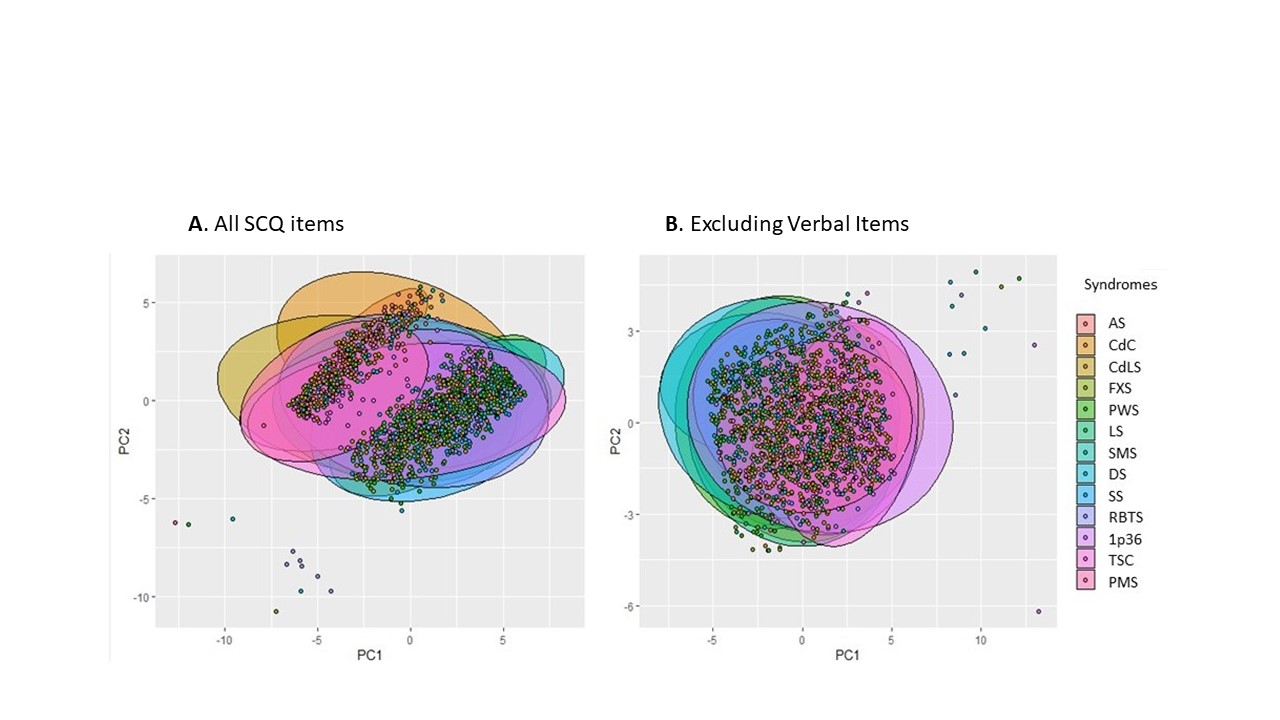
**

**Abbreviations:** AS - Angelman syndrome, CdCS - Cri du Chat syndrome, 1p36- 1p36 deletion syndrome, CdLS - Cornelia de Lange syndrome, FXS - fragile X syndrome, PWS – Prader-Willi syndrome, LS - Lowe syndrome, SMS – Smith-Magenis syndrome, DS – Down syndrome, SS- Sotos syndrome, RTS- Rubinstein-Taybi syndrome, TSC- tuberous sclerosis complex, PMS – Phelan-McDermid syndrome

**Notes:** The different colours represent the thirteen genetic syndromes.

**Correlations**

**Supplementary Table 1.** Correlations between the number of individuals assigned to each group and the post-hoc predicted probability

| **Number of individuals assigned to each group** | | | | | | | | | | | | | | |
| --- | --- | --- | --- | --- | --- | --- | --- | --- | --- | --- | --- | --- | --- | --- |
| **Predicted probability** |  | **AS** | **FXS** | **PWS** | **RTS** | **CdLS** | **DS** | **CdCS** | **1p36** | **LS** | **SMS** | **TSC** | **SS** | **PMS** |
|  | **AS** | **.989*** | - | - | - | - | - | - | - | - | - | - | - | - |
|  | **FXS** | -.135 | **.977**** | - | - | - | - | - | - | - | - | - | - | - |
|  | **PWS** | -.148 | .290 | **.965*** | - | - | - | - | - | - | - | - | - | - |
|  | **RTS** | -.116 | -.054 | -.029 | **.998**** | - | - | - | - | - | - | - | - | - |
|  | **CdLS** | .174 | .328 | .371 | -.098 | **.978**** | - | - |  | - | - | - | - | - |
|  | **DS** | -.140 | .178 | .693* | -.090 | .256 | **.979**** | - | - | - | - | - | - | - |
|  | **CdCS** | .302 | .258 | .135 | -.198 | .233 | .038 | **.967**** | - | - | - | - | - | - |
|  | **1p36** | .031 | .150 | .030 | -.349 | .349 | -.057 | -.040 | **.731*** | - | - | - | - | - |
|  | **LS** | .007 | .735* | .449 | -.120 | .562* | .350 | .228 | .239 | **.903**** | - | - | - | - |
|  | **SMS** | -.046 | .476* | .304 | -.074 | .450 | .228 | .155 | .180 | .569* | **.658*** | - | - | - |
|  | **TSC** | -.154 | .543* | .504* | -.185 | .582* | .376 | .050 | .471 | .688* | .609* | **.862**** | - | - |
|  | **SS** | -.195 | .804** | .661* | -.071 | .439 | .530* | .197 | .142 | .871* | .830** | .706* | **.963**** | - |
|  | **PMS** | .207 | .336 | .090 | -.325 | .560* | .002 | .029 | .585* | .474 | .393 | .489* | .322 | **.616*** |
|  | **n** | 154 | 297 | 278 | 102 | 199 | 135 | 75 | 41 | 89 | 54 | 83 | 35 | 40 |

**Abbreviations:** AS - Angelman syndrome, CdCS - Cri du Chat syndrome, 1p36 – 1p36 deletion syndrome, CdLS - Cornelia de Lange syndrome, FXS - fragile X syndrome, PWS – Prader-Willi syndrome, LS - Lowe syndrome, SMS – Smith-Magenis syndrome, DS – Down syndrome, SS- Sotos syndrome, RTS- Rubinstein-Taybi syndrome, TSC- tuberous sclerosis complex, PMS – Phelan-McDermid syndrome, n = sample size

**Notes: ****p<0.001, *p<= 0.05

**Supplementary Table 2**. SVM results after the addition of self-help skills (WESSEX) as an additional predictor

| **Frequency of assigned class for the thirteen genetic syndromes** | | | | | | | | | | | | | | |
| --- | --- | --- | --- | --- | --- | --- | --- | --- | --- | --- | --- | --- | --- | --- |
|  | **AS** | **CdCS** | **CdLS** | **FXS** | **PWS** | **LS** | **SMS** | **DS** | **SS** | **RTS** | **1p36** | **TSC** | **PMS** | **Total** |
|  | **n** | **n** | **n** | **n** | **n** | **n** | **n** | **n** | **n** | **n** | **n** | **n** | **n** | **n** |
| **AS** | **133** | 17 | 26 | 5 | 3 | 8 | 5 | 6 | 1 | 3 | 9 | 2 | 11 | 229 |
| **CdCS** | 3 | **24** | 2 | 0 | 0 | 1 | 1 | 0 | 0 | 0 | 2 | 0 | 0 | 33 |
| **CdLS** | 12 | 9 | **103** | 13 | 7 | 16 | 7 | 5 | 3 | 5 | 14 | 18 | 16 | 228 |
| **FXS** | 2 | 10 | 26 | **237** | 41 | 35 | 22 | 12 | 15 | 9 | 0 | 22 | 5 | 436 |
| **PWS** | 3 | 11 | 37 | 33 | **202** | 22 | 13 | 43 | 17 | 8 | 6 | 29 | 0 | 424 |
| **LS** | 0 | 0 | 0 | 0 | 0 | **6** | 0 | 0 | 0 | 0 | 0 | 0 | 0 | 6 |
| **SMS** | 0 | 0 | 0 | 0 | 0 | 0 | **4** | 0 | 0 | 0 | 0 | 0 | 0 | 4 |
| **DS** | 1 | 4 | 5 | 9 | 23 | 1 | 0 | **68** | 3 | 2 | 0 | 1 | 2 | 119 |
| **SS** | 0 | 0 | 0 | 0 | 0 | 0 | 2 | 0 | **0** | 0 | 0 | 0 | 0 | 2 |
| **RTS** | 0 | 0 | 0 | 0 | 2 | 0 | 0 | 1 | 1 | **75** | 0 | 1 | 0 | 80 |
| **1p36** | 0 | 0 | 0 | 0 | 0 | 0 | 0 | 0 | 0 | 0 | **10** | 0 | 0 | 10 |
| **TSC** | 0 | 0 | 0 | 0 | 0 | 0 | 0 | 0 | 0 | 0 | 0 | **10** | 0 | 10 |
| **PMS** | 0 | 0 | 0 | 0 | 0 | 0 | 0 | 0 | 0 | 0 | 0 | 0 | **1** | 1 |
| **Total n** | 154 | 75 | 199 | 297 | 278 | 89 | 54 | 135 | 40 | 102 | 41 | 83 | 35 | 1582 |
| **Accuracy**  **(%)** | 133/154 (86%) | 24/75 (32%) | 103/199 (52%) | 237/297  (80%) | 202/278  (73%) | 6/89  (7%) | 4/54  (7%) | 68/135  (50%) | 0/40  (0%) | 75/102  (74%) | 10/41  (24%) | 10/83  (12%) | 1/35  (3%) | 873/1582  (55%) |

**Abbreviations:** AS - Angelman syndrome, CdCS - Cri du Chat syndrome, 1p36 – 1p36 deletion syndrome, CdLS - Cornelia de Lange syndrome, FXS - fragile X syndrome, PWS – Prader-Willi syndrome, LS - Lowe syndrome, SMS – Smith-Magenis syndrome, DS – Down syndrome, SS- Sotos syndrome, RTS- Rubinstein-Taybi syndrome, TSC- tuberous sclerosis complex, PMS – Phelan-McDermid syndrome, n- sample size

| **Frequency of assigned class for the thirteen genetic syndromes** | | | | | | | | | | | | | | | |
| --- | --- | --- | --- | --- | --- | --- | --- | --- | --- | --- | --- | --- | --- | --- | --- |
|  | **AS** | **CdCS** | **CdLS** | **FXS** | **PWS** | **LS** | **SMS** | **DS** | **SS** | **RTS** | **ASD** | **1p36** | **TSC** | **PMS** | **Total** |
|  | **n** | **n** | **n** | **n** | **n** | **n** | **n** | **n** | **n** | **n** | **n** | **n** | **n** | **n** | **n** |
| **AS** | **137** | 20 | 25 | 7 | 4 | 9 | 6 | 6 | 1 | 3 | 0 | 7 | 3 | 8 | 236 |
| **CdCS** | 2 | **17** | 1 | 2 | 0 | 1 | 1 | 0 | 0 | 0 | 0 | 1 | 0 | 0 | 25 |
| **CdLS** | 9 | 9 | **98** | 8 | 3 | 13 | 9 | 4 | 2 | 2 | 5 | 7 | 11 | 11 | 191 |
| **FXS** | 2 | 12 | 29 | **196** | 33 | 24 | 12 | 12 | 13 | 4 | 23 | 3 | 20 | 1 | 384 |
| **PWS** | 1 | 8 | 30 | 26 | **187** | 14 | 13 | 42 | 15 | 7 | 12 | 3 | 19 | 5 | 382 |
| **LS** | 0 | 0 | 0 | 0 | 0 | **8** | 0 | 0 | 0 | 0 | 0 | 0 | 0 | 0 | 8 |
| **SMS** | 0 | 0 | 0 | 0 | 0 | 0 | **2** | 0 | 0 | 0 | 0 | 0 | 0 | 0 | 2 |
| **DS** | 1 | 3 | 8 | 8 | 31 | 4 | 0 | **64** | 2 | 2 | 1 | 2 | 6 | 2 | 134 |
| **SS** | 0 | 0 | 0 | 0 | 0 | 0 | 0 | 0 | **0** | 0 | 0 | 0 | 0 | 0 | 0 |
| **RTS** | 0 | 1 | 0 | 0 | 1 | 1 | 0 | 0 | 0 | **79** | 0 | 0 | 0 | 0 | 82 |
| **ASD** | 2 | 5 | 8 | 50 | 19 | 15 | 11 | 7 | 7 | 5 | **217** | 5 | 17 | 6 | 374 |
| **1p36** | 0 | 0 | 0 | 0 | 0 | 0 | 0 | 0 | 0 | 0 | 0 | **13** | 0 | 0 | 13 |
| **TSC** | 0 | 0 | 1 | 0 | 0 | 0 | 0 | 0 | 0 | 0 | 0 | 0 | **7** | 1 | 9 |
| **PMS** | 0 | 0 | 0 | 0 | 0 | 0 | 0 | 0 | 0 | 0 | 0 | 0 | 0 | **1** | 1 |
| **Total n** | 154 | 75 | 200 | 297 | 278 | 89 | 54 | 135 | 40 | 102 | 258 | 41 | 83 | 35 | 1841 |
| **Accuracy**  **(%)** | 137/154 (89%) | 17/75 (23%) | 98/199 (49%) | 196/297  (66%) | 187/278  (67%) | 8/89  (9%) | 2/54  (4%) | 64/135  (47%) | 0/40  (0%) | 79/102  (77%) | 217/258  (84%) | 13/41  (32%) | 7/83  (8%) | 1/35  (9%) | 1034/1841  (56%) |

**Supplementary Table 3.** SVM results after the addition of the ASD group

**Abbreviations:** AS - Angelman syndrome, CdCS - Cri du Chat syndrome, 1p36 – 1p36 deletion syndrome, CdLS - Cornelia de Lange syndrome, FXS - fragile X syndrome, PWS – Prader-Willi syndrome, LS - Lowe syndrome, SMS – Smith-Magenis syndrome, DS – Down syndrome, SS- Sotos syndrome, RTS- Rubinstein-Taybi syndrome, TSC- tuberous sclerosis complex, PMS – Phelan-McDermid syndrome, ASD- autism spectrum disorder, n- sample size

**Abbreviations:** AS - Angelman Syndrome, CdC - Cri du Chat, CdLS - Cornelia de Lange Syndrome, FXS - Fragile X Syndrome, PWS – Prader-Willi Syndrome, LS - Lowe Syndrome, SMS – Smith Magenis Syndrome, DS – Down Syndrome, SS- Sotos Syndrome, RBTS- Rubinstein-Taybi Syndrome, TSC- Tuberous Sclerosis Complex, PMS – Phelan-McDermid Syndrome, n- sample size
